# AFI-Net: Attention-Guided Feature Integration Network for RGBD Saliency Detection

**DOI:** 10.1155/2021/8861446

**Published:** 2021-03-30

**Authors:** Liming Li, Shuguang Zhao, Rui Sun, Xiaodong Chai, Shubin Zheng, Xingjie Chen, Zhaomin Lv

**Affiliations:** ^1^School of Information Science and Technology, Donghua University, Shanghai 201620, China; ^2^School of Urban Railway Transportation, Shanghai University of Engineering Science, Shanghai 201620, China

## Abstract

This article proposes an innovative RGBD saliency model, that is, attention-guided feature integration network, which can extract and fuse features and perform saliency inference. Specifically, the model first extracts multimodal and level deep features. Then, a series of attention modules are deployed to the multilevel RGB and depth features, yielding enhanced deep features. Next, the enhanced multimodal deep features are hierarchically fused. Lastly, the RGB and depth boundary features, that is, low-level spatial details, are added to the integrated feature to perform saliency inference. The key points of the AFI-Net are the attention-guided feature enhancement and the boundary-aware saliency inference, where the attention module indicates salient objects coarsely, and the boundary information is used to equip the deep feature with more spatial details. Therefore, salient objects are well characterized, that is, well highlighted. The comprehensive experiments on five challenging public RGBD datasets clearly exhibit the superiority and effectiveness of the proposed AFI-Net.

## 1. Introduction

RGBD salient object detection tries to utilize the pair of RGB and depth images to highlight the visually fascinating regions in RGBD scenarios. Especially with the fast progress of RGBD hardware sensing equipment, such as the traditional Microsoft Kinect, modern smart phones, the advanced time-of-flight camera, and the motion capturing system [1, 2], depth information can be acquired continently and has played an crucial role in many related areas, such as scene understanding [3], semantic segmentation [4], RGBD saliency detection [5], ship detection [6–8], traffic signs detection [9,10], image thumbnails [11], and hand posture detection [12]. Thus, RGBD saliency detection has also received considerable attention, and significant efforts [5, 13–23] have also been exerted on this research area.

However, RGBD saliency models mainly rely on hand-crafted features, such as contrast computation [5, 13], minimum barrier distance computation [22], and the cellular automata model [20]. The performance of these RGBD saliency models degrade largely when handling some complex RGBD scenes with attributions, including small salient objects, heterogeneous objects, cluttered background, and low contrast. This phenomenon can be attributed to the weak representation ability of hand-crafted features in RGBD saliency models. Fortunately, significant progress has been achieved in deep learning theories in the past few years. In particular, convolutional neural networks (CNNs), which provided high level semantic cues, have been applied in RGBD saliency detection successfully [24–35], such as the three stream structure in [34], the fluid pyramid integration in [35], and the complementary fusion in [28].

Though the performance of existing deep learning-based RGBD saliency models is encouraging, they still lose their efficiency when dealing with complex RGBD scenes. Thus, the performance in the area of RGBD saliency detection can still be improved. In addition, some fusion-based RGBD saliency models [5, 14, 15, 19, 27, 28, 32, 33] aim to integrate two modalities, namely, RGB and depth information, through early fusion, middle fusion, and result fusion. These models often result in cross-modal distribution gap or information drop, leading to performance degradation. Meanwhile, the attention mechanism [36] has been widely adopted in many saliency models [37–39], enhancing the saliency detection performance in RGB image scenes. Furthermore, boundary information has been applied in salient object detection [40, 41], providing more spatial details for salient objects.

Thus, this work proposes an innovative end-to-end RGBD saliency model, that is, attention-guided feature integration network (AFI-Net). AFI-Net can extract and fuse features and perform saliency inference. Specifically, our model first extracts multimodal and level deep features, with the pair of RGB and depth images as the input. Then, the attention module, where the attention mechanism [36] is adopted to generate the attention map, enhances the multilevel RGB and depth features, yielding enhanced deep features. Next, these enhanced features (originated from different modalities and levels) are fused hierarchically. Lastly, the RGB and depth boundary features, that is, low-level spatial details, and the integrated feature are combined to perform saliency inference, yielding a high-quality saliency map. Our model focuses on RGBD saliency detection, whereas the existing boundary-aware saliency models [40, 41] focus on performing saliency detection in RGB images.

More importantly, the key advantages of the AFI-Net are the attention model, which indicates the salient objects coarsely, and the boundary information, which provides more spatial details for features. Thus, we can characterize salient objects perfectly in RGBD scenes. The general contributions of AFI-Net are described as follows:

1. We propose AFI-Net to highlight the salient objects in RGBD images. The AFI-Net has three components, the extraction and fusion of features, and saliency inference.
2. To sufficiently utilize deep features from different modalities and levels, the attention module is employed to enhance deep features and guide the hierarchical feature fusion. Furthermore, the spatial details are further embedded in the saliency inference step to obtain accurate boundary details.
3. We perform exhaustive experiments on five public RGBD datasets, and our model achieves the state-of-the-art performance. The experiments also validate the effectiveness of the proposed AFI-Net.

## 2. Related Works

The pioneer effort [42] defined saliency detection using the center-surround difference mechanism, and succeeding works constructed many saliency models to detect salient objects in natural scene RGB images. Meanwhile, the research on RGBD saliency detection [43, 44] has also been pushed forward significantly in recent decades. Many RGBD saliency models exist, including heuristic models [5, 13–23] and deep learning-based models [24–35, 45], which have achieved encouraging performance. Following, we introduce some of the existing RGBD saliency models.

In [14], color contrast, depth contrast, and spatial bias are combined to generate saliency maps. In [5], luminance-, color-, depth-, and texture-based features are used to produce contrast maps, which are combined to compute for the final saliency map using weighted summation. In [15], the features maps computed by using region grouping, contrast, and location and scale are combined to conduct RGBD salient object detection. In [19], compactness saliency maps computed using color and depth information are aggregated into a saliency map via the weighted summation approach. In [20], color- and depth-based saliency maps are integrated and improved via the linear summation and cellular automata. In [24], various feature vectors, such as local contrast, global contrast, and background prior, are generated and fused to infer the saliency value of each superpixel.

With the wide deployment of CNNs, the performance of RGBD saliency models is significantly advanced. In [25], depth features are combined with appearance features using the fully connected layers, generating high-performance saliency maps. In [27, 28], a two-stream architecture is proposed with a fusion network to detect the complementarities of RGB and depth cues. In [31], two networks, namely, a master network and a subnetwork, are used to obtain deep RGB and depth features, respectively. In [29], RGBD salient object detection is performed using a recurrent CNN. In [35], multilevel features are fused and used to detect salient objects using a fluid pyramid network. In [33], two-stream networks interact to further explore the complementarity of multimodal deep features. In [32], a fusion module is employed to fuse the RGB and depth-based saliency results.

## 3. Methodology

First, the proposed AFI-Net is introduced in Section 3.1. Then, the feature extraction is presented in Section 3.2. Subsequently, feature fusion and saliency inference are described in Section 3.3. Finally, in Section 3.4, some implementation details are introduced.

### 3.1. Overall Architecture

**Figure 1:**
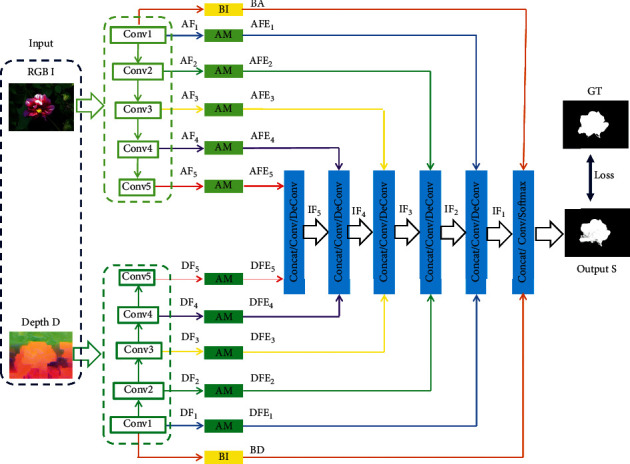
The architecture of the proposed AFI-Net.

Figure 1 summarizes our RGBD saliency model, AFI-Net, which includes a two-stream-based encoder (i.e., feature extraction), a single branch-based decoder (i.e., feature fusion), and saliency inference. Specifically, the entire network is constructed based on VGG-16 [46] with an end-to-end structure. RGB image **I** and depth map **D** are used as the input to AFI-Net. Here, the initial depth map is encoded into an HHA map **D** using [47]. Then, RGB image **I** and depth map **D** are sent to the two-stream network. Thus, we can obtain multilevel initial deep RGB features {**AF**_*i*_}_*i*=1_^5^ and deep depth features {**DF**_*i*_}_*i*=1_^5^, which correspond to the different convolutional blocks in each branch. Subsequently, a series of attention modules are deployed to enhance the initial deep features, yielding enhanced deep RGB features {**AFE**_*i*_}_*i*=1_^5^ and deep depth features {**DFE**_*i*_}_*i*=1_^5^. Next, the fusion branch is used to integrate the enhanced RGB and depth features hierarchically, and we can obtain integrated deep features {**IF**_*i*_}_*i*=1_^5^. Finally, the saliency inference module is employed to obtain a saliency map **S** by aggregating the boundary information, that is, the low-level spatial details. In Section 3.2, we elaborate the proposed RGBD saliency model, AFI-Net.

### 3.2. Feature Extraction

The feature extraction branch, namely, an encoder, is a two-stream network containing RGB and depth branches constructed based on VGG-16 [46]. Specifically, the RGB and depth branches have five convolutional blocks with 13 convolutional layers (kernel size =  3 × 3 and stride size = 1) and 4 max pooling layers (pooling size =  2 × 2 and stride size = 2). Here, considering the inherent difference of **I** and **D**, the RGB and depth branches have the same structure with different weights. Following this pipeline, we can obtain the initial multiple modalities and the multilevel features including the deep RGB features {**AF**_*i*_}_*i*=1_^5^ and the deep depth features {**DF**_*i*_}_*i*=1_^5^, as shown in Figure 1.

**Figure 2:**
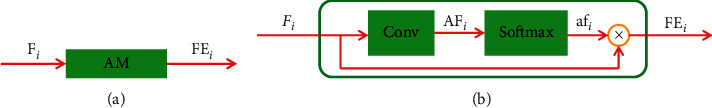
Architecture of attention module AM. (a) The thumbnail of AM. (b) The detailed configuration of AM.

On the basis of multimodal features {**AF**_*i*_}_*i*=1_^5^ and {**DF**_*i*_}_*i*=1_^5^, we first deploy the attention module, as shown in Figure 2, to further enhance the initial deep features. Formally, we denote each initial deep RGB feature **AF**_*i*_ or deep depth feature **DF**_*i*_ as **F**_*i*_ for convenience. According to Figure 2(b), attention feature **AF**_*i*_ is formulated as follows:

(1) $$\begin{array}{c} AF_{i}=\text{Conv}\left(F_{i}\right), \end{array}$$

where Conv denotes a convolutional layer. Then, we can compute the attention weight (**af**_*i*_(*w*, *h*)) at each spatial location using softmax as shown in Figure 2(b):

(2) $$\begin{array}{c} af_{i}\left(w,h\right)=\frac{e^{AF_{i}\left(w,h\right)}}{\sum_{\left(w^{\prime },h^{\prime }\right)\in \left(W,H\right)}e^{AF_{i}\left(w^{\prime },h^{\prime }\right)}}, \end{array}$$

where (*w*, *h*) denotes the spatial coordinates of attention feature **AF**_*i*_ and the width and height of **AF**_*i*_ are denoted as (*W*, *H*). Notably, ∑_(*w*′, *h*′)∈(*W*, *H*)_**af**_*i*_(*w*′, *h*′)=1.

After obtaining attention map **af**_*i*_, initial deep feature **F**_*i*_ should be selected, which is formulated as follows:

(3) $$\begin{array}{c} FE_{i}=DF_{i}^{*}af_{i}, \end{array}$$

where *∗* is the Hadamard matrix product in the channel direction and **FE**_*i*_ is the enhanced deep feature. Thus, we can generate the enhanced deep RGB features {**AFE**_*i*_}_*i*=1_^5^ and the enhanced deep depth features {**DFE**_*i*_}_*i*=1_^5^.

### 3.3. Feature Fusion and Saliency Inference

To integrate the enhanced RGB features {**AFE**_*i*_}_*i*=1_^5^ and the enhanced depth features {**DFE**_*i*_}_*i*=1_^5^, the fusion branch, that is, the decoder, is deployed to fuse the multimodal and level deep features hierarchically, as shown in Figure 1. Specifically, the hierarchical integration operation is performed as follows:

(4) $$\begin{array}{c} IF_{i}=\left\{\begin{array}{cc} H\left(\left[DFE_{i},IF_{i+1},AFE_{i}\right]\right), & i<5,\\ H\left(\left[DFE_{i},AFE_{i}\right]\right), & i=5, \end{array}\right. \end{array}$$

where *H* denotes the fusion and contains one convolutional layer and one upsampling layer, [.] denotes channel-wise concatenation, and **IF**_*i*_ is the *i*^th^ integrated deep feature.

According to the descriptions, we can obtain the first integrated deep feature (**IF**_1_). On basis of **IF**_*i*_, we aggregate it with the low-level spatial detail features, that is, the boundary information, to obtain a saliency map. Specifically, as shown in Figure 1, boundary information **BA** and **B**  **D** can be obtained from the bottom layer conv1-2 in the RGB and depth branches, respectively, by using a convolutional layer (1 × 1), that is, a boundary module (BI box marked in yellow). Subsequently, the saliency prediction is performed by using two convolutional layers (3 × 3) and one softmax layer. Thus, the saliency inference is written as follows:

(5) $$\begin{array}{c} S=\text{Fun}\left(\left[IF_{1},BA,B\;D\right]\right), \end{array}$$

where the RGBD saliency map is represented by **S**, [.] denotes the channel-wise concatenation operation, and Fun() refers to the convolutional layers and the one softmax layer.

### 3.4. Implementation Details

AFI-Net includes feature extraction, feature fusion, and saliency inference. Concretely, **D**_train_={(**I**_*n*_, **D**_*n*_, **GT**_*n*_)}_*n*=1_^*N*^ is the training dataset, where **I**_*n*_={**I**_*n*_^*j*^,  *j*=1,…, *N*_*p*_}, **D**_*n*_={**D**_*n*_^*j*^,  *j*=1,…, *N*_*p*_}, and **GT**_*n*_={**GT**_*n*_^*j*^,  *j*=1,…, *N*_*p*_} refer to the RGB image, the depth map, and the ground truth with *N*_*p*_ pixels, respectively. Here, subscript *n* is dropped, and {**I**, **D**} corresponds to each RGB image and depth map pair. Thus, the total loss can be written as follows:

(6) $$\begin{array}{c} ℒ\left(W,b\right)=-\beta \sum_{j\in GT_{+}}\log\;P\left(GT^{j}=1|I,D;W,b\right)\\ -\left(1-\beta \right)\sum_{j\in GT_{-}}\log\;P\left(GT^{j}=0|I,D;W,b\right), \end{array}$$

where the kernel weights and bias of the convolutional layers are denoted as **W** and **b**, respectively; **GT**_+_ and **GT**_−_ denote the salient objects and backgrounds, respectively; and *β* is the ratio of salient objects' pixels in **GT**, that is, *β*=|**GT**_+_|/|**GT**_−_|. Furthermore, *P*(**GT**^*j*^=1*| ***I**, **D**; **W**, **b**) is the saliency value of each pixel.

AFI-Net is implemented using the Caffe toolbox [48]. During the training phase, the parameters of the SGD algorithm, such as momentum, base learning rate, minibatch size, and weight decay, are set to 0.9, 10^−8^, 32, and 0.0001, respectively. Our total iterations are set to 25,000. Furthermore, the learning rate is divided by 10 at the beginning of each 12,500 iterations. The VGG-16 model is used to initialize the weights of the RGB and depth branches. The fusion branch is initialized by using the “msra” method [49]. In addition, the training data used by CPFP [35] is also employed to train our model. The training data contain 1400 pairs of RGB and depth images from NJU2K [16] and 650 pairs of RGB and depth images from NLPR [15]. Obviously, augmentation operations are also adopted, including rotation with angles 90°, 180°, and 270° and mirroring. Finally, the number of training samples is 10250. After the training phase, we can obtain the final model with 131.2 MB. During the test phase, the average processing time per 288 × 288 image is 0.2512 s.

## 4. Experiments

The public RGBD datasets and the comprehensive evaluation metrics are described in Section 4.1.  In Section 4.2, exhaustive quantitative and qualitative comparisons are performed successively. Lastly, the ablation analysis is presented in Section 4.3.

### 4.1. Experimental Setup

To validate the proposed AFI-Net, we perform comprehensive experiments on five challenging RGBD datasets, namely, NJU2K [16], NLPR [15], STEREO [13], LFSD [50], and DES [14]. NJU2K includes 2003 samples, which are captured from the Internet, daily routines, and so on. From the dataset, 1400 samples are employed for training and 485 samples for testing, that is, “NJU2K-TE.” NLPR was constructed by Microsoft Kinect, consisting of 1000 samples, and the salient objects in some samples are more than one. For training the AFI-Net, 650 samples are selected from NLPR to construct the training set, and 300 samples are selected from NLPR to build the testing set, that is, “NLPR-TE.” STEREO has 1000 samples, which are used as the testing set. LFSD and DES consist of 100 and 135 samples, which are all used as the testing set. All the datasets are equipped with pixelwise annotation. To compare the RGBD saliency models quantitatively, max *F*-measure (max *F*), *S*-measure (*S*) [51], mean absolute error (MAE), and max *E*-measure (max *E*) [52] are utilized in this paper.

*S*-measure considers the region aware value (*S*_*r*_) and the object aware value (*S*_*o*_) simultaneously, measuring the structural similarity between the ground truth and the saliency map. Referring to [51], the formulation is defined as follows:

(7) $$\begin{array}{c} S=\alpha *S_{o}+\left(1-\alpha \right)*S_{r}, \end{array}$$

where *α* is a balance parameter (here, we set it to 0.5).

*F*-measure is the weighted harmonic mean of precision and recall and is formulated as follows:

(8) $$\begin{array}{c} F_{\beta }=\frac{\left(1+\beta^{2}\right)\text{precision}\times \text{recall}}{\beta^{2}\text{precision}+\text{recall}}, \end{array}$$

where *β*^2^ is set to 0.3. Max *F*-measure could be obtained using different thresholds [0, 255].

*E*-measure denotes the enhanced-alignment measure considering the local details and the global information. Referring to [52], *E*-measure can be written as follows:

(9) $$\begin{array}{c} \xi =\frac{2\varphi_{\text{GT}}\left(x,y\right){}^{\circ}\varphi_{\text{FM}}\left(x,y\right)}{\varphi_{\text{GT}}\left(x,y\right){}^{\circ}\varphi_{\text{GT}}\left(x,y\right)+\varphi_{\text{FM}}\left(x,y\right){}^{\circ}\varphi_{\text{FM}}\left(x,y\right)}\\ E=\frac{1}{W\times H}\sum_{x=1}^{W}\sum_{y=1}^{H}f\left(\xi \right), \end{array}$$

where *f*(·) denotes the convex function, ° denotes the Hadamard product, and *ξ* is the alignment matrix.

MAE measures the difference between ground truth **GT** and saliency map **S**:

(10) $$\begin{array}{c} \text{MAE}=\frac{1}{W*H}\sum_{i=1}^{W*H}\left|S\left(i\right)-GT\left(i\right)\right|, \end{array}$$

where the obtained saliency maps are scaled to [0, 1], *W* is the width of the saliency map, and *H* denotes the height.

### 4.2. Comparison with the State-of-the-Art Models

A comparison is first made on NJU2K-TE, NLPR-TE, STEREO, LFSD, and DES between AFI-Net and nine state-of-the-art RGBD saliency models, namely, CDCP [23], ACSD [16], LBE [18], DCMC [19], SE [20], MDSF [21], DF [24], AFNet [32], and CTMF [27]. The traditional heuristic RGBD saliency models represented by the first six RGBD saliency models and the last three RGBD saliency models are CNNs-based RGBD saliency models. Here, the saliency maps of the other models are provided by the authors or obtained through the source codes. Next, the quantitative and qualitative comparisons are presented. Specifically, Table 1 shows the quantitative comparison results on five RGBD datasets. AFI-Net outperforms the nine state-of-art RGBD saliency models in terms of all the evaluation metrics.

**Figure 3:**
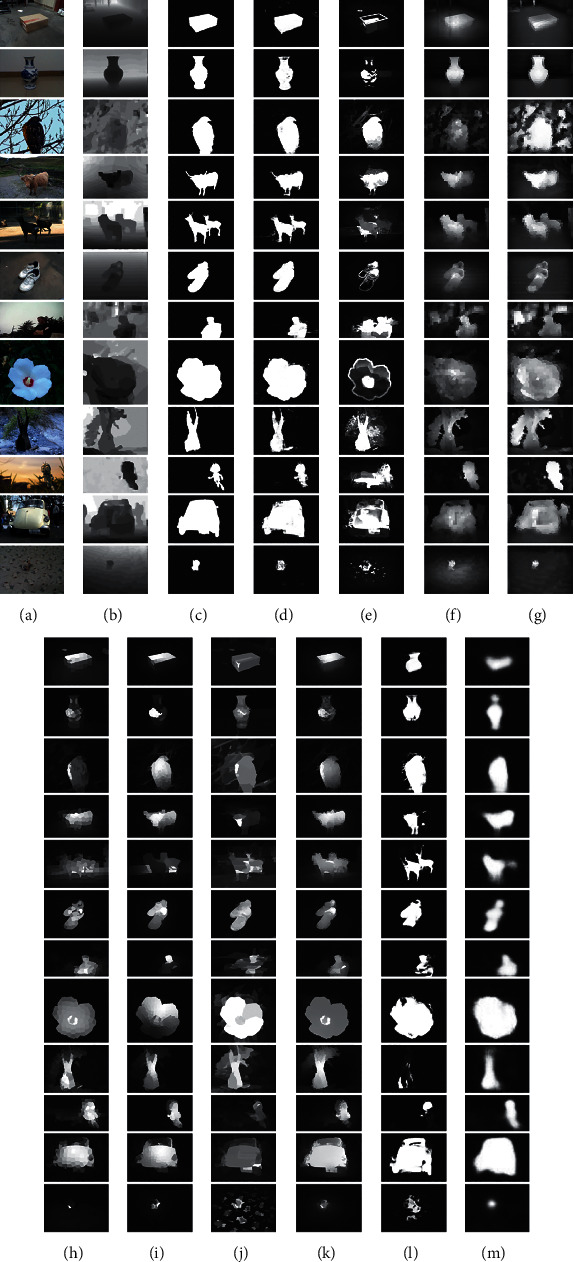
Qualitative comparison results on some challenging scenes. From left to right, (a) RGB, (b) depth, (c) GT, (d) ours, (e) CDCP, (f) ACSD, (g) LBE, (h) DCMC, (i) SE, (j) MDSF, (k) DF, (l) AFNet, and (m) CTMF.

Figure 3 presents the qualitative comparisons on some complex scenes. AFI-Net achieves superior performance over the nine state-of-the-art models. Specifically, the first example presents a box on the ground, where the box in the depth map is indistinctive. The other models shown in Figures 3(e)–3(m) falsely highlight some backgrounds and cannot pop-out the box completely. In the second example, the vase is a heterogeneous object, and its bottom is unclear in the depth map. Our model (Figure 3(d)) performs better than the other models though the top part is not popped-out completely. Like the first example, the third and the fourth examples not only show an unclear depth map but also present a cluttered background. Fortunately, our model still highlights the bird and the cow completely and accurately. The fifth and sixth examples show multiple salient objects. AFI-Net exhibits the best performance, as shown in Figure 3(d). In the seventh example, the man is in the image boundary, and its corresponding depth is also unclear. Under this condition, our model still performs better than the others though some backgrounds are also highlighted mistakenly. For the 8^th^ and 11^th^ rows, the salient objects occupy most regions of the images. Our model and the AFNet achieve comparable performance, as shown in Figures 3(d) and 3(m). The 9^th^ and the 10^th^ rows also show a cluttered background. Obviously, AFI-Net still exhibits the best performance as shown in Figure 3(d).

Generally, through the extensive comparison of AFI-Net and nine state-of-the-art models, we can demonstrate the proposed AFI-Net's effectiveness.

### 4.3. Ablation Studies

Here, the intensive study on some key components in AFI-Net is presented quantitatively and qualitatively. Specifically, the crucial points in AFI-Net include the attention module (AM) and the boundary module (BI), as shown in Figure 1. Therefore, we design two variants of our model, namely, AFI-Net without the attention module (denoted as “w/oA”) and the AFI-Net without the boundary module (denoted as “w/oB”). Correspondingly, we perform comprehensive comparisons between our model and the two variants.

**Figure 4:**
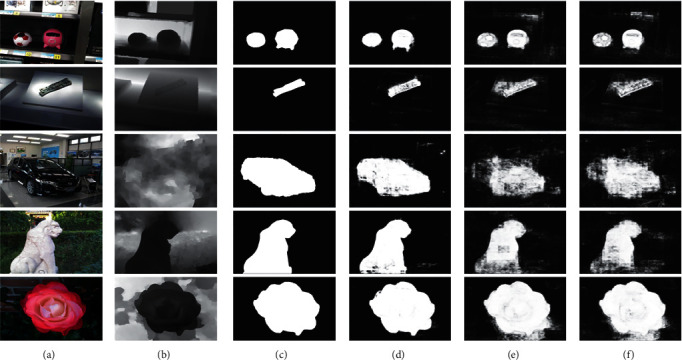
Qualitative comparisons of two variations of AFI-Net on some challenging examples. (a) RGB, (b) depth, (c) GT, (d) AFI-Net, (e) w/oA, and (f) w/oB.

First, the quantitative comparison results are presented in Table 2. Clearly, AFI-Net consistently outperforms the two variants, “w/oA” and “w/oB,” on two RGBD datasets. Secondly, the qualitative comparison results are presented in Figure 4. AFI-Net (shown in Figure 4(d)) performs better than the two variants (shown in Figures 4(e)) and 4(f)). The results of AFI-Net have well-defined boundaries and highlight the salient objects completely. In contrast, the two variants falsely highlight some backgrounds and cannot detect the salient objects completely.

Overall, the attention and boundary modules play an important role in AFI-Net, enhancing the deep features and equipping them with more spatial details. Meanwhile, the results clearly validate the rationality and effectiveness of the two components in the proposed AFI-Net.

### 4.4. Failure Case Analysis

**Figure 5:**
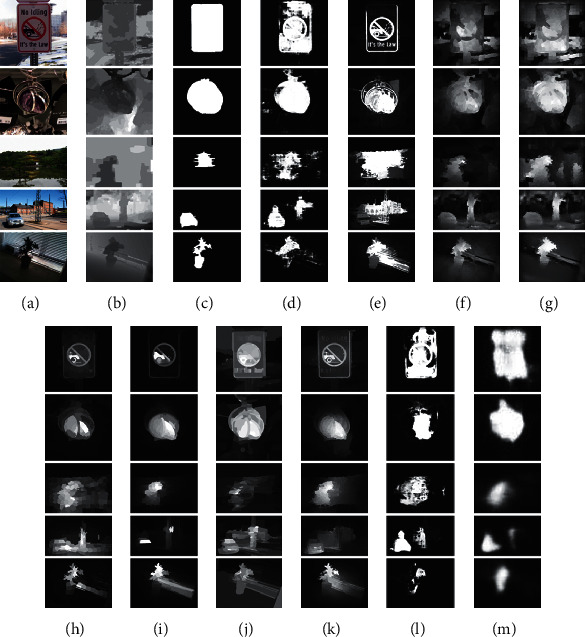
Some typical failure cases. From left to right, (a) RGB, (b) depth, (c) GT, (d) ours, (e) CDCP, (f) ACSD, (g) LBE, (h) DCMC, (i) SE, (j) MDSF, (k) DF, (l) AFNet, and (m) CTMF.

In the experiments, we demonstrate the effectiveness and rationality of the proposed AFI-Net. Particularly, Figure 3 shows the qualitative comparison between the proposed AFI-Net and the state-of-the-art saliency models, highlighting the effectiveness of the proposed AFI-Net. However, in some challenging images, our model cannot detect salient objects well, as shown in Figure 5(d). Specifically, in Figure 5, the first example shows a traffic sign, and all models fail to pop-out the salient object. The second example is a pendant, which is highlighted incompletely by most of the models. In the third example, which presents a pavilion, all models falsely pop-out the background regions. In the last two examples, the car and the pot culture cannot be detected accurately and completely. Although our model fails to highlight the salient objects of these examples, it can still pop-out the main part of the salient objects shown in Figure 5(d) better than the other models (shown in Figure 5(e))–5(m)) because our model contains an effective attention module, which covers the main parts of the salient objects. Generally, the research on RGBD saliency detection still faces many difficulties, and the research on the complex scene images is worthy of attention.

## 5. Conclusion

This work proposes an innovative RGBD saliency model AFI-Net, which can perform feature extraction, feature fusion, and saliency inference. Specifically, the generated initial multimodal and multilevel features are first promoted by a series of attention modules, which select the initial deep features and coarsely indicate the location of salient objects. Then, the hierarchical fusion branch is adopted to fuse the enhanced deep features, which are further combined with low-level spatial detail features (i.e., the boundary information) to perform saliency inference. Thus, the generated saliency maps can highlight salient objects and preserve sharp boundaries. The experiments results on five public RGBD datasets indicate that the proposed AFI-Net obtains superior performance over nine state-of-the-art models.

## Figures and Tables

**Table 1:** Quantitative comparisons on five public challenging RGBD datasets.

Metric		CDCP	ACSD	LBE	DCMC	SE	MDSF	DF	AFNet	CTMF	**AFI-net**
		[23]	[16]	[18]	[19]	[20]	[21]	[24]	[32]	[27]	**Ours**
NJU2K-TE	*S*↑	0.669	0.699	0.695	0.686	0.664	0.748	0.763	**0.772**	*0.849*	***0.854***
	max*F*↑	0.621	0.711	0.748	0.715	0.748	0.775	**0.804**	0.775	*0.845*	***0.853***
	max*E*↑	0.741	0.803	0.803	0.799	0.813	0.838	**0.864**	0.853	***0.913***	*0.903*
	MAE↓	0.180	0.202	0.153	0.172	0.169	0.157	0.141	**0.100**	*0.085*	***0.073***

NLPR-TE	*S*↑	0.727	0.673	0.762	0.724	0.756	**0.805**	0.802	0.799	*0.860*	***0.878***
	max*F*↑	0.645	0.607	0.745	0.648	0.713	**0.793**	0.778	0.771	*0.825*	***0.864***
	max*E*↑	0.820	0.780	0.855	0.793	0.847	**0.885**	0.880	0.879	*0.929*	***0.933***
	MAE↓	0.112	0.179	0.081	0.117	0.091	0.095	0.085	**0.058**	*0.056*	***0.045***

STEREO	*S*↑	0.713	0.692	0.660	0.731	0.708	0.728	0.757	**0.825**	*0.848*	***0.856***
	max*F*↑	0.664	0.669	0.633	0.740	0.755	0.719	0.757	**0.823**	*0.831*	***0.851***
	max*E*↑	0.786	0.806	0.787	0.819	0.846	0.809	0.847	**0.887**	*0.912*	***0.912***
	MAE↓	0.149	0.200	0.250	0.148	0.143	0.176	0.141	*0.075*	**0.086**	***0.068***

LFSD	*S*↑	0.717	0.727	0.729	0.753	0.692	0.700	**0.791**	0.738	**0.796**	***0.825***
	max*F*↑	0.703	0.763	0.722	**0.817**	0.786	0.783	*0.817*	0.744	0.791	***0.832***
	max*E*↑	0.786	0.829	0.797	0.856	0.832	0.826	**0.865**	0.815	*0.865*	***0.874***
	MAE↓	0.167	0.195	0.214	0.155	0.174	0.190	0.138	**0.133**	*0.119*	***0.097***

DES	*S*↑	0.709	0.728	0.703	0.707	0.741	0.741	0.752	**0.770**	***0.863***	*0.860*
	max*F*↑	0.631	0.756	**0.788**	0.666	0.741	0.746	0.766	0.728	***0.844***	*0.829*
	max*E*↑	0.811	0.850	**0.890**	0.773	0.856	0.851	0.870	0.881	***0.932***	*0.910*
	MAE↓	0.115	0.169	0.208	0.111	0.090	0.122	0.093	**0.068**	*0.055*	***0.050***

The three best results are denoted in bold-italic, italic, and bold fonts.

**Table 2:** Ablation analysis on NJU2K [16] and LFSD [50].

		w/oA	w/oB	AFI-Net
NJU2K-TE	*S*↑	0.824	0.826	**0.854**
	max*F*↑	0.816	0.820	**0.853**
	max*E*↑	0.881	0.884	**0.903**
	MAE↓	0.108	0.107	**0.073**

LFSD	*S*↑	0.803	0.798	**0.825**
	max*F*↑	0.803	0.796	**0.832**
	max*E*↑	0.847	0.843	**0.874**
	MAE↓	0.131	0.132	**0.097**

The best results are denoted in bold.

## Data Availability

Previously reported data were used to support this study and are available at https://doi.org/10.1109/cvpr.2012.6247708; https://doi.org/10.1145/2632856.2632866; https://doi.org/10.1007/978-3-319-10578-9_7; https://doi.org/10.1109/icip.2014.7025222; and https://doi.org/10.1109/cvpr.2014.359. These prior studies (and datasets) are cited at relevant places within the text as references [13, 14, 15, 16, 50].
